# Miscellaneous Items

**Published:** 1886-04

**Authors:** 


### MISCELLANEOUS ITEMS.

**In** Sunstroke, try artificial respiration.

**The** elder Coto was the Physician to his own family.

**The** Romans consulted the medical men of Epidamus 187 years B. C.

**Mithridates** the Great, discovered an antidote to certain poison thirty-six years B. C.

**The** Church Union will, after the first day of May, be published at No. 33 East 22nd Street.

**The** first physicians of Rome were the priests, and Sechagathus, a Greek, was the first Physician of Rome.

**Dr. Flint** died in this city March 13th, in the seventy-fourth year of his age—a wonderfully overrated man.

**Asclepiades**, the developer of the Atormic theory, practiced medicine in Rome 90 years B. C. He was also the inventor of the shower bath.

**To Our Exchanges.**—Please remember that our address is No. 75 and 77 Barclay St., and has been the same for more than two years. Will you be kind enough to address your papers there.

**Rhode Island** has adopted an amendment to her constitution prohibiting the sale of any intoxicatlng liquors. We were present as a reporter in the Rhode Island legislature when the first "Maine law" was passed, and telegraphed the news to Horace Greeley.

**Bananas** are very nutritious. Humboldt made an estimate showing that 44,000 pounds of bananas can be produced in soil that would raise 1,000 pounds of potatoes, and that where wheat enough for one man could be raised, bananas enough would grow for twenty-five.

**Prof.** Baird, Secretary of the Smithsonian Institute, very strongly recommends the boiling all water used for drinking. It certainly is the safest way. It is not all wells in the country that are pure by a great deal. The brightest looking water is often dangerous to health.

**The** rearing cattle in snow banks has proved expensive the last winter. When cattle are killed by the cold weather, it is harder for them, and less profitable to the owner. Perhaps the great cattle raisers will learn, some time, to give the poor animals comfortable quarters.

**Cures** of sciatica are reported as having taken place in Paris after a single application of Dr. Debove's method of freezing the skin above the painful parts with a spray of chloride of methyl. The operation is said to be applicable also to facial neuralgia.

**Analysis** of croton water shows that it is never fit to drink and all of it should be filtered before drinking. This is Prof. A. H. Brennan's advice after a very thorough analysis during every day in the summer and often in other months of the year. If persons would boil the water and then cool it, there would be much less injurious matter in it.

**An** Englishman who insulated his bedstead by placing underneath each post a broken off bottom of a glass bottle, says that he had not been free from rheumatic gout for fifteen years, and that he began to improve immediately after the application of the insulators. A local paper quoting the item, wisely adds: "There's many a fellow who could cure his gout if he would break off the bottoms of his glass bottles in time."

**Dr. Edson**, of the New York Health Department, has had the dye of some bright red stockings, which were made in Saxony, examined, and the analysis discovered that it contained arsenic and antimony. Both poison the skin, and what is known as antimony rash is produced by underclothing the dye of which is fixed with antimony. Children are the worst sufferers from antimony rash, as they are the most likely to wear the bright colors which contain the poison.

**The Garden Ennobling.**—The garden can, in a measure, satisfy the soul-longings for the beautiful. The smooth, grassy lawn with its handsome trees of varied form and foliage, the climbing vines that embower the porches, the flowers of every type and tint, these gratify wants that are felt by the higher man. As we make our homes beautiful by ornamenting our grounds with trees and plants, we are educating ourselves and our children in a higher life—a life distinct from that of the brute creation, and which is the glory of man, the lord of the earth.—*Vick's Magazine for February.*

**Turpentine Injection for Fistula.**—Cecchini reports having used turpentine in the radical cure of several varieties of fistulæ. He claims that the substance operates as a powerful anticeptis, produces granulating and can never do harm if a reasonable amount of care is exercised. He states several cases *Fistulæ in anis*, *Caries of Petrous Portion of Temporal Bone*, *Dental Fistula.* He has also used turpentine in the cure of carbuncles, and in post mortem wounds with most excellent success.

**Alcohol.**—What, think you, would be the effect upon the eye were we to drop even one drop of alcohol into it? It would immediately present all the symptoms of very strong poison, and for this very good reason, it *is* poisoned beyond any dispute. It has been touched with one of the most violent of poisons that is poisoning one-half the men in the country.

**The Insane Asylum.**—Pending the vote on the resolution of Dr. Gillespie, in regard to locating the West Tennessee Insane Asylum, one of the members ef the Tri-State Medical Society arose and said: "I favor a site near this city, for if I am to judge by the proceedings of this Society no place on earth has more imminent need of such an institution." The commissioners thought differently, however, and gave it to Bolivar.

**Chlorine Gas** is prepared by heating a mixture of four parts, by weight, of salt, one of oxyd of manganese, two of sulphuric acid and two of water. It is of a greenish color. It can be prepared in a saucer placed on a hot stove. School rooms, bed rooms and sick rooms should be disinfected frequently to drive out small pox, diphtheria, scarlet fever, and similar evils. Make the above described chlorine gas, and it will completely destroy all poisonous infectious influences.

**To Avoid Catching Cold.**—Accustom yourself to the use of sponging with cold water every morning on first getting out of bed. It should be followed with a good deal of rubbing with a wet towel. This gives tone to the skin, and enables it to resist the sudden changes of temperature. Sir Astley Cooper says: "The methods by which I have preserved my own health are temperance, early rising and sponging the body every morning with cold water immediately after getting out of bed, a practice which I have adopted for thirty years without ever catching cold."

**Physicians** at Reading, Pa., are puzzled over the freaks of Reuben Briner, age seven. The boy has a mania for fire and seeks to burn up everything he can lay his hands on. He formerly lived with his parents in Philadelphia, where he cremated his 18-months-old brother, and when punished for it threateded to kill the rest of the family. He was then sent to his grand-parents at Reading, where he has roasted several cats and burned up his shoes and other wearing apparel, and has made attempts to set fire to buildings. Is this a case that requires imprisonment, or have the sins of the parents been visited upon the poor, unfortunate child?

**Cauliflower.**—Cauliflower is of all northern vegetables the most nutritious, containing sixty per cent. of gluten, and sometimes more.

**Disinfection of Clothing** is best accomplished by baking in a heat exceeding that of boiling water. An effectual heat is 250 degrees, which will not scorch or change the color of the fabric. This plan is strongly recommended for infected bedding.

**Vegetable Remedies.**—Spinach acts as a diuretic; dandelion as a tonic and laxative; asparagus as a blood-cleaner; tomatoes as a chólegogue; beets and turnips are said to be tonic; Onions, garlic and leek are stimulating and narcotic; the red onion acts as narcotic in insomania and neuralgia.—*Medical Summary.*

**Deodorisers** may be chosen from any of the following:—Chlorine, chloride of lime, chloride of zinc, nitrate of lead, freshly-burned charcoal, sulphate of iron, and sulphate of lime. These are used by mixing them with the matter of fluid to be deodorized.

If liberally done with either of them, the offensive smell will be destroyed. The two last are the best; a quantity of either thrown into a cess-pool will render the matter entirely scentless in a few hours.

**Cocaine in the Treatment of Whooping Cough.**—In a pamphlet recently published, Dr. Moncorvo has advocated the employment of cocaine as an adjunct to the treatment of the malady by resorcin, for which Dr. Moncorvo stands the sponsor. The upper parts of the larynx are first to be mopped with the one per cent. solution of resorcin, and then a ten per cent solution of the hydrochlorate of cocoaine is applied. The cocoaine should be used frequently.

**Who Owns a Prescription.**—The Supreme Court of Massachusetts has lately made a decision about who owns a prescription: "The question before the Court seems to be very simple, indeed. A patient applies to a physician and receives from him certain advice, for which he tenders a fee. The physician hands a piece of paper to the patient, purporting to be a *written order* for certain goods, called drugs, which order is filled by a merchant or apothecary. The payment of the fee, and the delivery of the goods, or drugs, terminates the verbal contract, and the druggist keeps the prescription as evidence that the *contract* has been fulfilled, so far as he is concerned. The druggist can, if he so *please*, on his own responsibility, renew the drugs, for he is but a merchant, and has a *perfect right* to sell drugs to anyone and in any shape.

**A Singular Case.**—Dr. Walker Y. Corol, of 162 West Thirty-fourth street, sent recently to sanitary headquarters, the photograph of a child born without brains, and which lived a whole day. The record in the Bureau of vital statistics show that the child was born Christmas day, F. L. Nelson, blacksmith, being the father. It was a well formed child with the exception that it had no brain. Dr. Corol wrote on the malformation that the mother had lived last spring in a haunted house, in West Thirty-seventh street, between Tenth and Eleventh Avenues. The house is one of two, built together for private use by two brothers, one of whom died while the house was being built. Afterwards the house was finished, and occupied by a woman who was a Spiritualist, who died there. It seems that the Doctor has an idea that this has some influence in producing the curious defect. We fail to see any sort of connection in the case that would account for the lack of brains. We should much prefer to know the condition of the mother and father, and the constant manner of her treatment during pregnancy.

**An Old Woman's Remedies.**—L. Mitchell sends the following to the *Journal of Reconstruction:* "When the busy practitioner, the overworked business man or the worn-out journalist finds himself completely out of order, his muscles unnerved and his nerves unstrung, his digestion upset and his glands running away with him, let him take a sleeping car and go into the far country. He will find his mother or his aunt, or somebody else's mother or aunt; this is the person to cure him. The remedies of the motherly person are as follows:

| | | |
|---|---|---|
| Rhubarb, | Ginger, | Fruit, |
| Soda, | "Composition," | Ammonia, |
| Peppermint, | Sweet oil, | Fresh Calamus, |
| Catnip, | *Fresh Air*, | Fresh Wintergreens, |
| Camphor, | *Cheerfulness*, | Wild Cherry, |
| Licorice, | *Exercise*, | Elderberry wine, |
| Wormwood, | *Rest*, | Milk and cream, |

Is not this excellent? Can not such a regime be systematized and employed by the regular profession in proper cases?

**D. F. Wright, M. D.**, on the Bulletin of the Tennessee Board of Health, has an excellent article on the subject of eggs, from which we make the following extract:

"A portion of the yolk placed in the field of the microscope is found to consist of globular bodies, which are oil or fat globules, some much smaller bodies (the yolk corpuscles), and a viscid fluid; the oil globules contain a fluid very similar in its constitution to animal fats, though with a difference of some importance. Animal fat consists of margarine oleine and stearine, which are compounds of margaric oleic and stearic acids with the well known organic base entitled glycerine ; the oil of the yolk globules differs from the fat mainly in omitting the stearine, which is the constituent which contributes most to the solidity of the fat. This omission probably renders the yolk fat more digestible than, for instance, beef, mutton or pork fat. Each fat globule is enclosed in an envelope from the fluid in which the globules and corpuscles float.

But besides the oil these globules contain a mineral substance of indispensable importance, the phosphate of lime. The corpuscles as well as the fluid in which they float are largely supplied with a substance which has been called by the chemists "*vitellin*," but which is simply a mixture of two better known substances, *casein* and *albumen*, the former being largely predominant. These belong to a group of organic materials constituting by far the greater part of the animal body; they have been called the *protein* compounds, but Prof. Huxley's term protoplasm is now their accepted name. *Casein*, *albumen* and *fibrine* are the principal members of the group ; they are all made up of the four elements carbon, hydrogen, oxygen and nitrogen combined in the same proportion, and the members only differ in regard to the presence of minute quantities of sulphur and phosphorous in various proportions. No food which does not contain one or more of tnese substances is capable of sustaining animal life, and, from its predominant occurrence in milk and eggs, casein is inferred to be the one most readily convertible into animal tissues. These corpuscles and their surrounding fluid also contain a considerable amount of sulphur, as is shown by their blackening a silver spoon with a deposit of silver sulphide.

The occurrence of two different phosphates in the egg offers pregnant suggestions toward the purpose in view. The same two occur in widely different departmenes of the human system. The mono basic phosphate of the yolk corresponds exactly with the phosphate found so largely in the human brain and nervous system, and the tribasic phosphate of the white is identical with the bone earth or mineral constituent of the bones."

To sum up the whole article, he makes eggs to be a most excellent article of diet, a statement to which we fully agree, but they must be taken when they are fresh and not kept for weeks or months "packed" in any form yet discovered; or sent hundreds of miles on a railroad to market. We speak of fresh eggs.

**French Lavender Flowers.**—"The best lavender flowers," said a wholesale druggist of Greenwich street, "come from France. The flowers are small and blue in color, and are mixed with small stems as big as small pins. They are very fragrant, and when rubbed on the hand leave a delicate and pleasant odor. I'll guarantee that nine out of ten persons who use lavender water in their toilet have not the vaguest idea what lavender flowers look like. Speaking of lavender water reminds me that, although it is so called, the perfume used is not lavender water but lavender extract.

"How does it come to this country?

"In packages of about three hundred pounds each. It is not very expensive. We sell it at 12 cents a pound."

"In what form is it imported other than the flower?"

"The extract and the essential oil. The latter is a volatile oil upon which the odor depends. The ordinary oil sells from $1.75 to $8 a pound. Witchman's oil of lavender, which gets its name from Witchman's garden in London, costs $4.25 an ounce. An ounce of fairly good oil will perfume four or five gallons of alcohol."

"Is lavender not also used for medicinal purposes?"

"It is a remedy for nervous debility and one or two other complaints. It is very pleasant to the taste, and for this reason is often mixed with other medicines. The best lavender extract is made in Germany. In fact, many of the French perfumers have all their extracts made in Germany, because they make them better there than anywhere else."

**Turnips and Carrots.**—These roots are about of equal value as food. The dried meal of either contains gluten, starch, and sugar, and is very nutritious. That of the turnips is rather superior, and is almost equal to Indian corn, being only deficient in fat. Attempts have been made to manufacture a palatable meal from dried turnips, but the disagreeable taste of the root so clings to the meal as hitherto to have rendered it objectionable.

**Essentials of Food.**—The materials of the food of man, containing the necessary elements, are derived from the mineral, vegetable and animal kingdoms. The vegetable kingdom, however, is the great source of food to man and animals, as it is in the cells of the plant that the elements undergo those chemical changes which fix them for food. Though animal food is more nutritious, it is only because it is a condensed form of the vegetable substances from which the flesh is derived, as the substances supplied by animal food are identical with those obtained from plants.

**Snakes in the Stomach.**—We copy the following from a recent issue of the Reno (Nev.) *Gazette:* "The wife of Lem Allen, a prominent citizen of Churchill county, has been an invalid for a long time. Occasionally her sufferings were great, and recently her ailments were most serious, such as to cause her friends to almost lose hopes of her surviving. While laboring under the most acute pain, accompanied with symptons of inflammation of the stomach and bowels, the most heroic medicines were used. After a time four snakes of the water species were taken from her. One was quite three feet in length, another about twenty inches, and two about eight inches. The powerful medicines used poisoned them, and evidently they remained dead in the stomach some little time. How they were taken into the stomach and survived is a question. For quite a while the lady seriously complained of a peculiar sensation as if something was creeping around within, little thinking there was any reality in it. Since the serpents were unwittingly poisoned she has recovered rapidly, and bids fair to soon enjoy her wonted health.

**What Oleomargarine Is.**—We are in receipt of the extract from the Report made to the legislature on the qualities of Oleomargarine. It is worse as a mixture, than we had supposed. Here are the compounds which enter into the greatest substitute for butter. We presume it may be made with less of the injurious ingredients, but it is not so made according to the report: Indigestible, insoluble, and even putrescent substances are employed in the patent processes of manufacture, among which may be enumerated sugar of lead, bisulphate of lime, saltpetre, borax, boracic acid, salicylic acid, benzoic acid, orris root, cotton seed oil, vegetable oils, bitaric acid, bicarbonate of soda, nitrate of potassia, glycerine, capcylic acid, cuparic acid, alum, capcic acid, sulphate of soda, cows' udders, commercial sulphuric acid, pepsin, salsoda, tallow, lard, sea salt, farinaceous flour, butyric ether, caustic potash, carbonic acid, sulphuric acid, castor oil, curcumine, chalk, slippery elm bark, caul, oil of sesame, oil of sunflower seeds, olive oil, turnip seed oil, bromo chloralum, chlorate of potash, niter, oil of sweet almonds, oil of peanuts, peroxide of manganese, stomach of pigs, sheep or calf; nitrate of soda, bennie oil, gastric juice, mustard seed oil, nitric acid, dry blood, albumen, sugar, butyric acid, bicarbonate of potash, chloride of sodium, caustic soda, corn starch, and various coloring matters. The "coloring matter" is something that is largely practiced by all manufacturers of butter and is not, of course, considered "dangerous," but some of the substances are decidedly so.

**Mr. H. C. Harvey** mentions the case of a cat that has become quite blind from cataract, but is still able to do mousing and to make journeys to and from home.

**The Wall Paper Peril.**—"Chemist" writes to the *Advertiser:* "I may be able to throw a little light on the question. Formerly greens were the only arsenical colors, but within the last ten years an extensive use has been made by paper stainers of a compound of arsenic and glycerine. That enters now into almost all colors, and especially into the most delicate tinted papers. A richness and a softness is secured by this compound that defies competition. Tinted book papers of the most delicate tints and of the highest cost owe their richness and delicacy of tint to the glycerine and arsenic solution. Hence the importance of putting wall papers and tinted papers under proper surveillance.

**Health Alphabet.**—The Ladies' Sanitary Asssociation, of London, gives the following simple rules for keeping health:

A—s soon as your are up shake blanket and sheet;
B—etter be without shoes that sit with wet feet;
C—hildren, if healthy, are active not still;
D—amp bed and damp clothes will both make you ill;
E—at slowly and always chew your food well;
F—reshen the air in the house where you dwell;
G—arments must never be made too tight;
H—omes should be healthy, airy and light;
I—f you wish to be well, as you do I've no doubt,
J—ust open the windows before you go out;
K—eep the rooms always tidy and clean;
L—et dust on the furniture never be seen;
M- uch illness is caused by the want of fresh air;
N—ow, to open the windows be ever your care;
O—ld rags and old rubbish should never be kept;
P—eople should see that their floors are well swept;
Q—uick movements in children are healthy and right;
R—emember the young cannot thrive without light;
S—ee that the cistern is full to the brim;
T—ake care that your dress is all tidy and clean;
U—se your nose to find if there be a bad drain;
V—ery sad are the fevers that come in its train;
W—alk as much as you can without feeling fatigue;
X—erxes could walk full many a league;
Y—our health is your wealth which your wisdom must keep;
Z—eal will help a good cause, and the good you will reap.

**Chronic Enlargement of Tonsils.**—In answer to Doctor Gaff's Inquiry in regard to hypodermic injections in the above named condition: Dr. Beresford, in the October number of the *Medical Advocate*, says: By the use of a strong solution of tannic acid injected two or three times a week, with the dailey use of a gargle of the same, the knife need never be resorted to."

I have used the above treatment in the case of a young man, æt. about twenty years, with good success. I make an application of muriate cocaine before inserting the needle, and used the injection every three days.—*Cal. Med. Journal.*

**Damp Linen** —Damp linen is sufficient to account for frequent colds, consumption, and premature death of a whole family; and where the mischief having not take that direction, it is developed in the form of rheumatism, which does once set in from that cause, is generally incurable. All body-linen, shortly before putting on, should be made dry by a good fire. Whenever that is impossible, it is a good plan to put the linen between the blankest of your bed all night, in a position to get plenty of heat. Those who have experienced no signal evidence of the mischief of damp linen are apt to be careless on the subject; but the carelessness will inevitably entail its punishment, which is likely to accumulate insidiously until it is too late.

**Who Use Bread.**—Baked loaves of bread, it appears, are unknown in many parts of South America and of Italy, and throughout the agricultural districts of Roumania. In the villages of the Obersteiermark, not very many miles from Vienna, bread is never seen, the staple food of the people being *sterz*, a kind of porridge made from ground beechnuts, which is taken at breakfast with fresh or curdled milk, at dinner with broth or fried lard, and with milk again for supper. This *sterz* is also known as *heiden*, and takes the place of bread, not only in the Steiermark, but in Carinthia and in many parts of the Tyrol. In the North of Italy, the peasantry live chiefly on *polenta*, a porridge made of boiled maize. The *polenta*, however, is not allowed to granulate like Scotch porridge or like the Austrian *sterz*, but is boiled into a solid pudding, which is cut up and portioned out with a string. It is eaten cold as often as it is hot, and is in every sense the Italian peasant's daily bread. A variation of the *polenta*, called *mamaliga*, is the national dish of Roumania. The *mamaliga* is like the *polenta* in that it is made of boiled maize; but it is unlike the latter in one important respect, as the grains are not allowed to settle into a solid mass, but are kept distinct, after the fashion of oatmeal porridge.

